# Suspected Thymoma as the Initial Presentation of Graves’ Disease: A Report of a Rare Case and Comprehensive Literature Review

**DOI:** 10.7759/cureus.111785

**Published:** 2026-06-30

**Authors:** Madison McNulty, Dinesh Nirmal, Marija Kaljevic, Joseph Fares, Aastha Baral, Maksim Agaronov, Samy I McFarlane

**Affiliations:** 1 Internal Medicine, State University of New York Downstate Health Sciences University, Brooklyn, USA; 2 Endocrinology, State University of New York Downstate Health Sciences University, Brooklyn, USA; 3 Pathology, State University of New York Downstate Health Sciences University, Brooklyn, USA

**Keywords:** anterior mediastinal mass, graves´disease, hyperthyroidism, neoplastic mass, percutaneous biopsy, thymic epithelial tumor, thymic hyperplasia, thymoma, thyrotropin receptor-stimulating antibodies

## Abstract

Graves’ disease (GD) is an autoimmune endocrinopathy characterized by thyrotropin receptor-stimulating antibodies that lead to hyperthyroidism. In addition to thyroid dysfunction, patients with GD may develop extrathyroidal manifestations, including thymic hyperplasia (TH), which often presents as an incidental anterior mediastinal mass that typically regresses with treatment of hyperthyroidism. In contrast, a thymoma is a rare thymic epithelial tumor that may present with similar imaging findings but requires distinct diagnostic and therapeutic management. The coexistence of GD and thymoma is exceedingly rare. This report describes the case of a 35-year-old female patient with newly diagnosed GD who was incidentally found to have an anterior mediastinal mass. Imaging and subsequent percutaneous biopsy were highly suspicious for thymoma, illustrating the diagnostic challenge of distinguishing TH from thymoma in patients with GD. This case underscores the importance of maintaining a broad differential for persistent thymic enlargement in hyperthyroidism, ensuring neoplastic masses are not misattributed to benign TH.

## Introduction

The thymus, derived from the third and fourth pharyngeal pouches, is an anterior mediastinal organ essential for T-lymphocyte maturation and central immune tolerance. Disruption of normal thymic function can lead to the development of autoreactive T cells and subsequent autoimmune disease. Graves’ disease (GD) is a common autoimmune endocrinopathy mediated by stimulatory autoantibodies against the thyrotropin receptor (TSH-R), resulting in thyrocyte proliferation and excess thyroid hormone production [1].

In addition to thyroid dysfunction, patients with GD may develop extrathyroidal manifestations, including thymic abnormalities [2]. Benign thymic hyperplasia (TH) is the most common of these and is thought to result from both direct autoimmune stimulation and the trophic effects of excess thyroid hormone on thymic tissue. This thymic enlargement is often incidentally identified on chest imaging as an anterior mediastinal mass and characteristically regresses following the successful treatment of hyperthyroidism [3].

Thymoma, a neoplasm arising from thymic epithelial cells, can also present as an anterior mediastinal mass on imaging. Although its association with GD is rare, thymoma should be considered when the mass persists or fails to regress following treatment of hyperthyroidism. Differentiating benign TH from thymoma is clinically important, as management strategies differ substantially and have significant implications for patient outcomes [3].

This report describes a rare case of an anterior mediastinal mass, highly suspicious for thymoma based on chemical-shift MRI and core biopsy.. This case highlights the diagnostic challenges in differentiating TH from thymoma and underscores the importance of maintaining a broad differential diagnosis when evaluating thymic enlargement in patients with GD.

## Case presentation

A 35-year-old female patient presented with a two-week history of progressively worsening shortness of breath and intermittent hemoptysis. The patient had no known past medical, autoimmune, or family history, was not taking any medications, and denied any history of smoking. She denied known risk factors for deep vein thrombosis (DVT) and had no known exposure to tuberculosis (TB), and her review of systems was notably negative for weight loss, tremor, heat intolerance, palpitations, ophthalmopathy, menstrual changes, and compressive symptoms.

On presentation, vital signs were notable for tachycardia with a heart rate of 127 beats per minute (bpm) and a regular rhythm. Physical examination revealed a palpable, nontender goiter. The patient was not in acute respiratory distress; however, pulmonary auscultation demonstrated mild bilateral wheezing. Given the patient’s symptoms and tachycardia, computed tomography pulmonary angiography (CTPA) was performed to evaluate for pulmonary emboli (PE).

The CTPA, while negative for PE, incidentally revealed a 6.3 cm anterior mediastinal mass (Figure 1). Further characterization via dedicated chest magnetic resonance imaging (MRI) revealed a bilobed anterior mediastinal mass in the paracentral prevascular region measuring approximately 5.1 x 2.4 x 2.8 cm. The mass demonstrated homogenous T1- and T2-weighted intermediate signal with mild contrast enhancement. Notably, chemical-shift MRI was performed to evaluate for microscopic fat; however, there was no change in signal intensity between the in-phase and opposed-phase sequences. Quantitative metrics such as chemical shift ratio (CSR), signal intensity index (SII), and fat-fraction values were not calculated in the institutional radiologic report. There was a clear fat plane with the sternum and no pleural involvement or adenopathy (Figure 2).

**Figure 1:**
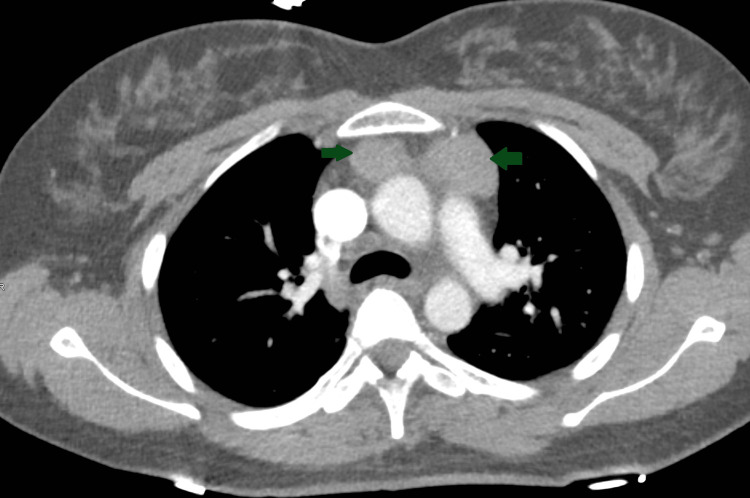
Axial view of a chest CT angiogram revealing a prominent, bilobed soft tissue mass in the anterior mediastinum. The two green arrows highlight the individual lobes of the mass.

**Figure 2:**
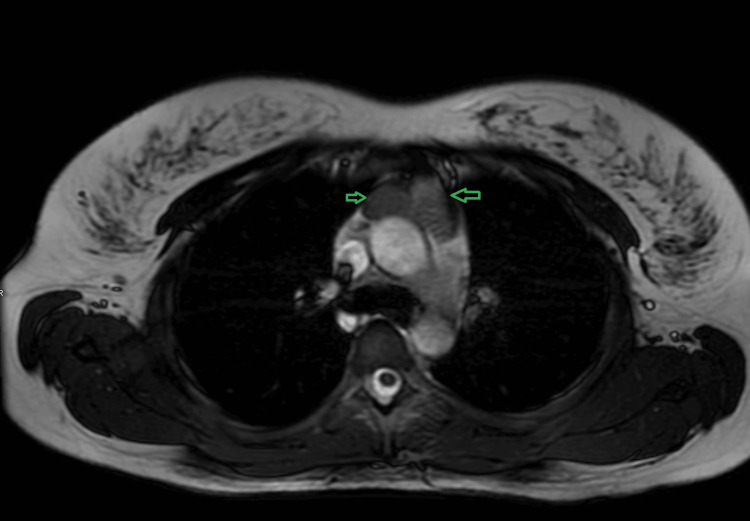
Axial T2-weighted chest MRI revealing a prominent, bilobed soft tissue mass in the anterior mediastinum. The two green arrows highlight the individual lobes of the mass.

Thyroid function tests revealed overt hyperthyroidism with a suppressed thyroid-stimulating hormone (TSH) level of < 0.005 µlU/mL and an elevated free thyroxine (T4) of 3.75 ng/dL. The patient was promptly initiated on methimazole and propranolol for suspected thyrotoxicosis and was closely monitored for the potential development of thyroid storm, particularly following iodinated contrast exposure from the initial CTPA.

Further evaluation demonstrated positivity for TSH-R antibodies (34.40 IU/L) and thyroid peroxidase antibodies (42.7 IU/mL), confirming a diagnosis of GD. During the remainder of her hospitalization, the patient remained hemodynamically stable with preserved mentation and reported improvement in her symptoms. Her thyroid function tests gradually improved but did not fully normalize during the admission. Evaluation for concurrent paraneoplastic myasthenia gravis (MG) was negative, with an acetylcholine receptor (AChR) antibody level of < 0.07 nmol/L (Table 1).

**Table 1:** Summary of thyroid function and autoantibody panel TSH: thyroid-stimulating hormone

Lab Test	Result	Reference Value
TSH level	<0.005 µIU/ml	0.27-4.2 µIU/ml
-Free thyroxine (T4)	3.75 ng/dl	0.8 to 1.8 ng/dL
TSH-receptor antibodies	34.40 IU/L	<0.9 IU/L
Thyroid peroxidase antibodies	42.7 IU/ml	<34 IU/ml
Acetylcholine receptor antibodies	<0.07 nmol/L	<0.30 nmol/L

One week after hospital discharge, the patient underwent an interventional radiology-guided transthoracic percutaneous core biopsy of the anterior mediastinal mass. Histological evaluation with hematoxylin and eosin staining revealed a biphasic proliferation composed of numerous immature-appearing lymphocytes admixed with clusters of bland epithelial cells possessing oval nuclei and inconspicuous nucleoli, alongside occasional Hassall corpuscle-like structures (Figure 3). Flow cytometry and immunohistochemistry confirmed the lymphoid component was a population of immature T-lymphocytes (thymocytes) positive for TdT, CD3, and CD5 (Figure 4). These thymocytes were dispersed within a lacy network of epithelial cells, demonstrating positive immunostaining for p63 and broad-spectrum cytokeratin (AE1/AE3), while staining negative for epithelial membrane antigen (EMA) (Figure 5). Although the combined biphasic morphology and immunophenotypic profile were highly suspicious for a lymphocyte-rich thymoma, the scant nature of the core biopsy specimen made the overall architecture difficult to assess, precluding a definitive diagnosis.

**Figure 3:**
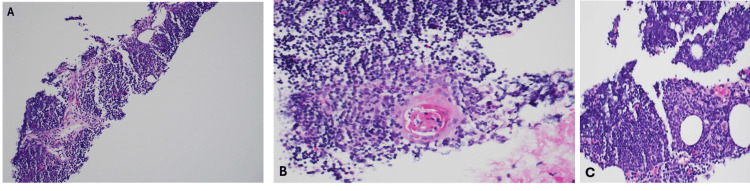
Histopathological features of the anterior mediastinal mass. (A) Hematoxylin and eosin (H&E) stain at 200x original magnification demonstrating a dense, lymphocyte-predominant infiltrate from the core biopsy specimen. (B) H&E stain at 400x original magnification, highlighting a Hassall corpuscle-like structure. (C) H&E stain at 400x original magnification, revealing a biphasic proliferation composed of numerous immature-appearing lymphocytes admixed with clusters of bland epithelial cells possessing oval nuclei and inconspicuous nucleoli.

**Figure 4:**
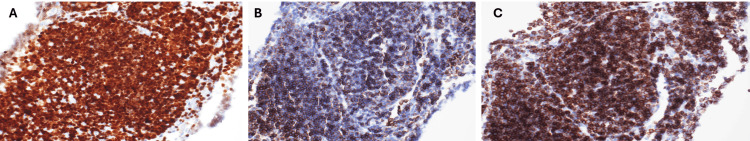
Immunohistochemical characterization of the lymphoid population. Immunohistochemical staining highlights the abundant lymphoid component, with cells demonstrating positivity for (A) terminal deoxynucleotidyl transferase (TdT), (B) CD5, and (C) CD3 (original magnification 400× for all). This immunophenotypic profile is consistent with an infiltrate of immature T-lymphocytes (thymocytes).

**Figure 5:**
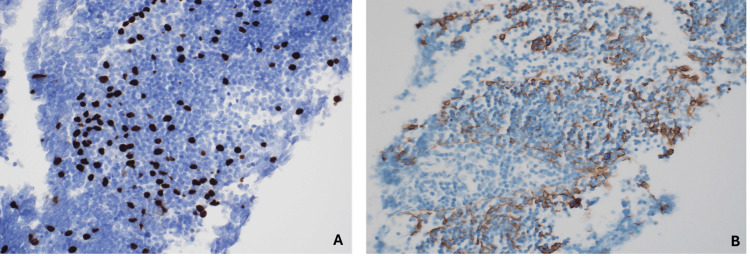
Immunohistochemical characterization of the epithelial component. The epithelial cell population is highlighted by positive immunostaining for (A) p63 and (B) broad-spectrum cytokeratin (AE1/AE3), revealing a lacy network of epithelial cells dispersed among the thymocytes (original magnification 400× for both). Immunostaining for epithelial membrane antigen was negative (not shown).

Additionally, to evaluate the patient's presentation of shortness of breath and intermittent hemoptysis, a cardiopulmonary workup was initiated. Electrocardiogram (ECG) demonstrated normal sinus rhythm with no ischemic abnormalities, and cardiac troponin was negative. CTPA showed no evidence of a PE but revealed findings compatible with pulmonary edema. Laboratory analysis was notable for an elevated pro-B-type natriuretic peptide of 280 pg/mL (reference range: 1-125 pg/mL). Given the structurally normal echocardiogram and an absence of alternative etiologies, the pulmonary edema was attributed to high-output heart failure secondary to thyrotoxicosis. Her shortness of breath resolved during the hospital stay without indication for repeat imaging.

The case was reviewed by a multidisciplinary tumor board, which recommended a cardiothoracic surgery-guided excisional biopsy to establish a conclusive diagnosis and guide definitive management. As cardiothoracic surgery services were unavailable at our institution, the patient was referred to an outside center for the procedure. At the time of this report, she has not returned for further follow-up.

## Discussion

Introduction to the diagnostic dilemma

The discovery of an anterior mediastinal mass during the clinical evaluation of a patient presenting with hyperthyroidism introduces a profound diagnostic and therapeutic dilemma. The anterior mediastinum, which has been recently redefined and reclassified by the International Thymic Malignancy Interest Group (ITMIG) as the prevascular compartment, represents a complex anatomical space bounded by the sternum anteriorly, the pericardium posteriorly, the thoracic inlet superiorly, and the diaphragm inferiorly. This compartment primarily contains the thymus gland, an array of lymph nodes, adipose tissue, and portions of the left brachiocephalic vein, making it the primary site for a wide spectrum of pathologies [4]. When a space-occupying lesion is identified in this region, the differential diagnosis is notably broad, spanning from entirely benign developmental anomalies and hyperplastic phenomena to rare neuroendocrine or autoimmune syndromes and aggressive, life-threatening malignancies such as thymic epithelial tumors, lymphomas, and malignant germ cell tumors [5,6].

Within the specific clinical context of GD, an autoimmune disorder characterized by the pathological overproduction of thyroid hormones driven by circulating TSH-R autoantibodies, the presence of an anterior mediastinal mass most frequently represents benign TH [7]. However, distinguishing this benign, fundamentally reversible hyperplastic response from a true neoplastic process, such as a thymoma, is of paramount importance [8]. The imperative to accurately differentiate these entities stems from the profound implications for patient management. Historically, patients presenting with profound thyrotoxicosis and a concomitant mediastinal mass were frequently subjected to immediate, invasive diagnostic procedures, including core needle biopsies, fine-needle aspirations, or even median sternotomy for complete thymectomy, driven by the fear of an underlying malignancy [9].

Such invasive surgical approaches carry substantial inherent morbidity and mortality risks, the most severe being the precipitation of a thyroid storm. Thyroid storm is a catastrophic, hypermetabolic state induced by surgical stress in an inadequately controlled hyperthyroid patient, carrying a mortality rate that can range between approximately 10-30%, depending on severity and access to care [7]. Consequently, advancements in high-resolution cross-sectional imaging, particularly the utilization of chemical-shift MRI, alongside an enhanced understanding of the intricate immunological pathophysiology connecting the thyroid gland and the thymus, have entirely revolutionized the contemporary standard of care [10]. A conservative, medically managed approach is now widely accepted in the absence of suspicious radiologic features [11].

This report reviews the literature on the coexistence of GD and thymic abnormalities. We detail the pathophysiological mechanisms driving thymic enlargement and outline the key radiological and histopathological criteria necessary to differentiate benign TH from thymic neoplasms.

The epidemiological and clinical landscape: literature search and case comparisons

A comprehensive review of the existing medical literature demonstrates that while macroscopic, space-occupying thymic enlargement is considered relatively rare in standard clinical practice, microscopic and histological abnormalities of the thymus are remarkably common in the setting of GD. Autopsy records and historical histological studies suggest that up to 38% of patients presenting with active thyrotoxicosis possess some measurable degree of thymic abnormality, ranging from simple lymphoid infiltration to profound follicular hyperplasia [12]. Despite this high histological prevalence, the true incidence of massive, macroscopic TH remains largely unknown. This statistical blind spot exists primarily because cross-sectional chest imaging is not routinely indicated, nor is it cost-effective, for the standard clinical evaluation of uncomplicated GD [13]. Consequently, the vast majority of documented cases emerge purely as incidental findings when advanced thoracic imaging is ordered to evaluate unrelated or overlapping symptoms, such as atypical dyspnea, suspected pulmonary embolism, non-specific chest pain, or fatigue [7].

The Rarity of True Thymic Neoplasms in Graves' Disease

To effectively structure a comparative literature review for a clinical case report, one must analyze the statistical probability of encountering a true thymoma in a patient with GD. By the year 2014, comprehensive literature reviews had cataloged approximately 107 well-documented cases of massive TH associated specifically with GD. Within this extensive cohort of patients presenting with significant anterior mediastinal masses, actual thymic malignancies were identified and confirmed in only four patients. This profound statistical rarity underscores a critical clinical paradigm that must guide initial management: when an anterior mediastinal mass is identified in the setting of active, uncontrolled GD, the statistical probability overwhelmingly favors benign hyperplasia over a malignant thymic epithelial tumor [7].

The coexistence of a true thymoma with GD, in the absence of other autoimmune phenomena, is an exceptional clinical event. Thymic epithelial tumors (TETs) are intrinsically linked to autoimmunity, but they are far more commonly associated with other paraneoplastic autoimmune disorders. Most notably, MG is observed in up to 46% of all patients diagnosed with a thymoma [9]. Other frequent paraneoplastic associations include pure red cell aplasia (accounting for up to 30% of TET-associated cytopenias), autoimmune hemolytic anemia, and Good's syndrome, a profound adult-onset immunodeficiency characterized by hypogammaglobulinemia [14].

While it is well-established that patients suffering from MG frequently exhibit coexisting autoimmune thyroid diseases, including Hashimoto's thyroiditis and GD, the direct, isolated overlap of a thymoma solely with GD without concurrent MG is sparsely documented in the global literature [9]. A recent systematic review of the literature highlighted the extreme rarity of this specific phenomenon, noting that prior to a newly reported case of an ectopic cervical thymoma in a GD patient in 2023, only three cases of anterior mediastinal thymomas coexisting directly with GD had been conclusively documented and published [14].

Other extensive literature reviews have noted isolated, single-case occurrences of T-cell lymphoblastic lymphoma and exceptionally rare instances of thymic lymphoepithelial carcinoma presenting in patients with sustained hyperthyroidism [10]. These malignant outliers, while statistically rare, serve as a necessary and sobering warning to the clinician. While TH is by far the most probable diagnosis, the possibility of a highly aggressive neoplastic etiology cannot be dismissed entirely without rigorous, methodical radiological evaluation and extended clinical follow-up (Table 2) [15].

**Table 2:** Epidemiological comparison of thymic pathologies in Graves' Disease References: [7,9,14]

Epidemiological Feature	Thymic Hyperplasia in Graves' Disease	Thymoma in Graves' Disease
Histological Prevalence	Present in up to 38% of thyrotoxic patients	Exceptionally rare
Macroscopic Presentation	~107 massive cases documented by 2014	Only 3 mediastinal, 1 cervical ectopic documented
Primary Autoimmune Link	TSHR antibodies and hyperthyroidism	Acetylcholine receptor antibodies (Myasthenia Gravis)
Associated Paraneoplastic Syndromes	None typically present	Pure red cell aplasia, Good's syndrome, hypogammaglobulinemia
Clinical Trajectory	Reversible with antithyroid medications	Progressive, requires surgical resection and potential chemotherapy

The pathophysiology of thymic hyperplasia in autoimmune thyrotoxicosis

The clinical association between GD and thymic enlargement was first documented in the surgical literature by Dr. William Halsted in 1914, yet the precise molecular and cellular mechanisms governing this relationship have only recently been elucidated through advanced immunological research [16]. Current evidence supports a complex, dual-pathway model involving both direct hormonal trophic effects driven by excess thyroid hormones and autoimmune-mediated cellular proliferation driven by specific autoantibodies [17].

To thoroughly understand these mechanisms, one must first categorize TH into its two distinct histological and structural subtypes: true TH and lymphoid follicular hyperplasia. True hyperplasia is characterized anatomically by a symmetric, proportional enlargement of the thymus gland, maintaining the normal microscopic architecture while exhibiting a massive increase in both epithelial and lymphoid elements. This results in a total glandular volume and weight that far exceeds the age-adjusted upper limit of normal. True hyperplasia is often considered a physiological stress response, frequently observed in clinical practice following recovery from severe systemic insults such as intensive cytotoxic chemotherapy, prolonged high-dose corticosteroid use, or extensive thermal burns, a phenomenon often termed rebound hyperplasia [7].

In sharp contrast, lymphoid follicular hyperplasia is characterized by the dense proliferation of highly active lymphoid follicles and germinal centers specifically within the thymic medulla, frequently occurring without a massive increase in the overall macroscopic dimensions of the gland [7]. This subtype is fundamentally linked to chronic autoimmune stimulation and dysregulation [11]. In the specific setting of GD, however, the pathological lines often blur, and both true structural hyperplasia and active lymphoid follicular hyperplasia can seamlessly coexist within the same gland, severely complicating both the macroscopic radiological presentation and the microscopic interpretation [18].

The Hormonal Trophic Overstimulation Hypothesis

The first major pathophysiological mechanism driving TH relies on the direct trophic influence of excess circulating thyroid hormones [16]. The human thymus is not a static organ; it is highly dynamic and exquisitely responsive to the systemic endocrine environment. In states of profound, unmanaged thyrotoxicosis, massively elevated circulating levels of T4 and triiodothyronine (T3) exert a direct, stimulatory effect on the thymic microenvironment. These hormones bind to specific nuclear receptors within the thymus, directly stimulating the rapid proliferation of both thymic cortical epithelial cells and medullary lymphoid follicles [19].

This hormone-dependent pathogenesis is heavily supported by longitudinal clinical observations. In many documented case reports, the rapid clinical onset of severe thyrotoxicosis perfectly mirrors the rapid, measurable volumetric expansion of the thymus gland. Conversely, the subsequent restoration of a normalized, euthyroid state through the meticulous administration of thionamides (such as methimazole, carbimazole, or propylthiouracil) or through definitive surgical total thyroidectomy leads to predictable, measurable thymic involution. The observation that thymic volume reliably decreases in tandem with the reduction of circulating T3 and T4 levels provides robust clinical evidence that the hyperthyroid state is a primary driver of thymic cellular expansion [17].

The Immunological Autoantibody Cross-Reactivity Hypothesis

The second, and arguably more complex, mechanism involves the direct cellular action of circulating autoantibodies. GD is fundamentally driven by the pathological generation of TSH-R antibodies. These autoantibodies bind to and mimic the action of physiological TSH, prompting the thyroid gland to synthesize and release hormones uncontrollably [8]. Crucially, advanced immunohistochemical research has demonstrated that thyrotropin receptors are not exclusively confined to the follicular cells of the thyroid gland; they are also widely expressed on human thymic epithelial cells [12].

When TSH-R autoantibodies circulate in high titers during active GD, they do not solely target the thyroid. They concurrently bind to and activate these extrathyroidal thyrotropin receptors located within the thymic architecture. This aberrant immunological cross-reactivity creates a localized, highly active inflammatory and proliferative environment within the prevascular compartment. The activation of these thymic TSH-Rs induces rapid thymic epithelial cell proliferation, contributing significantly to the overall glandular enlargement. The direct correlation between serum TSH-R antibody titers and overall thymic volume has been rigorously documented in multiple clinical tracking studies [12]. In these studies, a near-perfect linear relationship exists between the gradual normalization of autoantibody titers following immunosuppressive or antithyroid therapy and the corresponding volumetric reduction in thymic size, strongly suggesting that TSH-R autoantibodies possess a direct pathogenic role in driving thymic hyperplasia [20].

Furthermore, patients suffering from active GD with concurrent TH frequently demonstrate significantly elevated plasma levels of the soluble interleukin-2 receptor (sIL-2R). While sIL-2R is traditionally utilized in clinical oncology as a reliable tumor marker for tracking malignant lymphomas, its marked elevation in the context of GD signifies profound, systemic T-cell activation and widespread immune dysregulation [12]. Following the initiation of targeted antithyroid medical therapy or the execution of a total thyroidectomy, the rapid attenuation of plasma sIL-2R levels coincides directly and predictably with the radiological regression of the hyperplastic thymus mass [17]. This dual-mechanism model-hormonal overstimulation driving epithelial and lymphoid growth combined with direct autoantibody receptor activation driving targeted cellular proliferation-provides a comprehensive explanation for why TH is a near-ubiquitous, albeit frequently asymptomatic and subclinical, feature of severe autoimmune thyrotoxicosis [16].

Diagnostic imaging and radiological differentiation

Given the extraordinarily high morbidity associated with unnecessary thoracic surgery, alongside the catastrophic risk of precipitating a thyroid storm during general anesthesia, achieving a definitive, non-invasive diagnosis is an absolute clinical imperative [7]. The differentiation between a benign, reversible hyperplastic process and a highly malignant thymic neoplasm relies heavily on the utilization of advanced cross-sectional imaging modalities. These imaging techniques must go beyond mere morphological assessment to fundamentally evaluate the microscopic tissue architecture, vascularity, and intracellular lipid content of the identified mass. When standard imaging remains equivocal, only then should histopathological analysis through invasive biopsy be considered [21].

CT Morphological Characteristics

Contrast-enhanced CT (CECT) of the chest is universally employed as the initial imaging modality used to identify, measure, and anatomically map the location of an anterior mediastinal mass [5]. When evaluating a newly discovered lesion in the prevascular compartment, the radiologist constructs a differential diagnosis initially based on the predominant attenuation values of the tissue, categorized broadly into fat density, water density, soft tissue density, or calcification density [7].

In the specific context of a patient with known GD, a hyperplastic thymus typically presents on a CT scan as a diffuse, homogeneous, soft-tissue density enlargement [18]. The hyperplastic gland characteristically retains its normal bilobed, triangular, or "arrowhead" configuration, meticulously preserving smooth, straight, and well-defined outer margins [7]. Crucially, the radiological attenuation of the hyperplastic mass is roughly equivalent to that of the surrounding chest wall musculature, and there is an absolute and conspicuous absence of complex cystic changes, necrotic hypodense cores, or macroscopic calcifications [22]. The normal mediastinal fat planes surrounding the great vessels and pericardium remain entirely preserved, with no evidence whatsoever of structural invasion [21].

Conversely, thymic epithelial tumors (thymomas and thymic carcinomas) present with starkly divergent CT characteristics. Thymomas frequently present as unilateral, heavily lobulated, or globular masses that may demonstrate highly heterogeneous contrast enhancement patterns. Unlike the smooth preservation seen in hyperplasia, neoplastic lesions frequently exhibit internal hypodense regions indicative of necrotic or cystic degeneration, alongside the presence of either punctate or coarse calcifications [7]. In more advanced Masaoka-Koga stages, these tumors will demonstrate frank obliteration of surrounding fat planes and direct invasion into the pericardium, pleura, or the major prevascular structures, confirming their malignant nature (Table 3) [22].

**Table 3:** Differentiating CT characteristics of anterior mediastinal masses References:  [7,22]

Radiological Feature	Benign Thymic Hyperplasia	Thymic Epithelial Tumor (Thymoma)
General Morphology	Preserved bilobed, triangular, or "arrowhead" shape	Globular, asymmetrical, or lobulated mass
Border Characteristics	Smooth, straight, well-defined contours	Irregular, nodular, lacking distinct encapsulation
CT Attenuation Pattern	Homogeneous soft tissue density (isodense to muscle)	Heterogeneous density with variable enhancement
Internal Architecture	Normal microscopic fat infiltration observed	Often features necrosis or complex cystic degeneration
Macroscopic Calcifications	Uniformly absent	Frequently present (punctate, linear, or coarse)
Local Invasion	Strict preservation of adjacent fat planes	Frequent invasion of pericardium, pleura, or great vessels

The Gold Standard: Chemical Shift MRI

While computed tomography is excellent for initial spatial mapping and identifying gross structural invasion, Chemical shift MRI is a highly sensitive and specific imaging technique for non-invasively differentiating benign thymic hyperplasia from true thymic neoplasms [11]. The profound diagnostic utility of this technique relies not on gross morphology, but on evaluating the microscopic, intracellular composition of the tissue [23].

The normal, healthy thymus gland, as well as the hyperplastic thymus gland seen in GD, inherently contains a highly specific mixture of lymphoid tissue, epithelial tissue, and abundant microscopic fat cells (adipocytes) scattered uniformly throughout the interstitial connective tissue [24]. True thymic tumors, however, are composed of dense, solid, proliferating neoplastic epithelial cells and reactive lymphocytes that entirely physically displace and obliterate this normal microscopic adipose network, leaving the tumor utterly devoid of normal interstitial fat [25].

Chemical shift MRI capitalizes on this fundamental histological difference by utilizing T1-weighted gradient-echo sequences to acquire matched "in-phase" and "opposed-phase" (out-of-phase) images. The physics underlying this technique dictates that water protons and fat protons precess at slightly different resonant frequencies within the magnetic field. By meticulously timing the echo acquisitions, the signals from water and fat protons can be made to be additive on the in-phase images and entirely subtractive on the opposed-phase images [26]. When an MRI voxel contains a relatively equal mixture of both water and microscopic fat, as is universally the case in benign TH, there is a dramatic, visually apparent drop in signal intensity on the opposed-phase image compared to the in-phase image. Because solid thymic epithelial tumors completely lack this microscopic fat, their overall signal intensity remains stubbornly unchanged, or even slightly increased, between the two acquisition phases [23].

To move beyond qualitative visual assessment and establish highly reproducible diagnostic criteria, radiologists extract two primary quantitative metrics from these CS-MRI sequences: the Chemical Shift Ratio (CSR) and the Signal Intensity Index (SII) [24].

The chemical shift ratio: The CSR is a metric that compares the signal intensity (SI) of the thymus gland to the signal intensity of the adjacent paraspinal muscle across both imaging phases. The paraspinal muscle is utilized because it does not contain microscopic fat and thus serves as a highly stable, internal reference standard [26]. 

Extensive radiological validation has determined that a CSR value of 0.7 or lower indicates a profound signal drop relative to the muscle, confirming the abundant presence of normal microscopic fat infiltration and strongly correlating with a diagnosis of benign thymic hyperplasia [23]. Conversely, a CSR of 1.0 or higher indicates a complete absence of signal drop, virtually ensuring the presence of a solid thymic tumor devoid of fat [11]. While highly effective, the CSR does possess a recognized limitation: values falling between 0.8 and 0.9 are considered radiologically indeterminate and must be carefully correlated with patient age and other morphological features to derive a conclusion [23]. The formula for calculation is: $CSR = \frac{(SI_{thymus, opposed} / SI_{muscle, opposed})}{(SI_{thymus, in-phase} / SI_{muscle, in-phase})}$. 

Conversely, a CSR of 1.0 or higher indicates a complete absence of signal drop, virtually ensuring the presence of a solid thymic tumor devoid of fat [11]. While highly effective, the CSR does possess a recognized limitation: values falling between 0.8 and 0.9 are considered radiologically indeterminate and must be carefully correlated with patient age and other morphological features to derive a conclusion [23].

The signal intensity index:** **To circumvent the indeterminate zones of the CSR, the SII measures the direct percentage of absolute signal loss strictly within the thymic tissue itself, utilizing the formula: $SII = \frac{(SI_{in-phase} - SI_{opposed-phase})}{SI_{in-phase}} \times 100$.

The SII has proven in large-scale clinical trials to be a significantly more sensitive and reliable diagnostic metric than the CSR, particularly in younger adult populations where a higher baseline cellularity might cause some overlap in CSR values [27]. Rigorous prospective studies have established that an SII cutoff greater than 8.92% (frequently rounded to 9% in clinical practice) yields remarkable sensitivity and specificity for distinguishing hyperplastic thymus tissue from solid thymic tumors, approaching 100% in some studies [24].

In modern radiological practice, the calculation of a high SII effectively eliminates the need for an invasive diagnostic biopsy by unequivocally confirming the presence of normal interstitial adipose tissue scattered among the lymphoid elements [27]. Furthermore, emerging MRI techniques utilizing advanced multi-echo sequences to calculate precise fat fraction (FF) percentages have shown that a mean FF greater than 4.78% yields over 95% sensitivity and 100% specificity for diagnosing hyperplasia, further cementing MRI as the definitive diagnostic tool in the management of these patients (Table 4) [28].

**Table 4:** Chemical shift MRI quantitative metrics for differentiating mediastinal masses References: [23,24,28]

Quantitative MRI Metric	Calculation Methodology	Validated Cutoff for Hyperplasia	Validated Cutoff for Thymoma	Clinical Diagnostic Reliability
Chemical Shift Ratio (CSR)	Ratio of thymic SI to paraspinal muscle SI	≤ 0.7	≥1.0	High, but features indeterminate diagnostic zones between 0.8 and 0.9
Signal Intensity Index (SII)	Percentage drop in absolute thymic signal	> 8.92%	< 4.0%	Extremely High (100% sensitivity and specificity at established cutoff)
Mean Fat Fraction (FFmean)	Multi-echo Dixon quantification of fat	> 4.78%	< 4.78%	Exceptional (95.1% sensitivity, 100% specificity)

Histopathological differentiation: hyperplasia versus neoplasm

When high-resolution imaging results remain frustratingly equivocal, or when a mass displays definitively aggressive morphological features on CT, direct tissue sampling becomes a clinical necessity. However, the utilization of fine needle aspiration (FNA) and percutaneous core needle biopsy (CNB) for the evaluation of anterior mediastinal masses carries profound and well-documented diagnostic limitations [29,30].

Thymic epithelial neoplasms are exceedingly rare pathologies that are heavily dependent on complex, broad architectural features for accurate World Health Organization (WHO) classification [30]. The WHO classification system categorizes thymomas based on the microscopic morphology of the neoplastic epithelial cells and the relative proportion of reactive lymphocytes, dividing them into Types A, AB, B1, B2, and B3, alongside frank thymic carcinomas (Type C) [29].

The Diagnostic Peril of Core Needle Biopsy

Type B1 and B2 thymomas are particularly problematic for small-core biopsies because they are overwhelmingly classified as "lymphocyte-rich" tumors. In a Type B1 thymoma, the neoplastic epithelial cells are sparse, widely scattered, and completely overwhelmed by a dense background of reactive lymphocytes. When an FNA or a small-gauge CNB extracts tissue from a B1 or B2 thymoma, the microscopic field is entirely flooded with these benign, reactive T-lymphocytes [29,31].

In a limited tissue sample lacking a broad architectural context, this sea of lymphocytes can easily be misdiagnosed by even an experienced pathologist as reactive lymphoid hyperplasia, benign thymic hyperplasia secondary to GD, or even a low-grade lymphoma, resulting in a devastating false-negative diagnosis for a malignant neoplastic process [29]. The modern surgical trend toward utilizing increasingly minimally invasive, smaller-gauge biopsy needles severely exacerbates this sampling error, as the interpreting pathologist fundamentally requires a broad architectural view of the organotypic organization to render a definitive and safe diagnosis [30]. Because prediction of malignancy is nearly impossible on purely cytological cellular features obtained from an FNA, incisional or complete excisional surgical biopsies remain the gold standard for definitive tissue diagnosis [32].

Immunohistochemistry and the "Lacy Pattern"

When a sufficient, broad tissue sample is successfully obtained-either via a large surgical biopsy or a complete thymectomy-advanced immunohistochemical staining is indispensable for definitively differentiating a highly active hyperplastic thymus from a lymphocyte-rich B-type thymoma [29]. The entire diagnostic focus rests on mapping the spatial distribution, physical morphology, and complex networking of the thymic epithelial cells suspended among the dense lymphoid stroma [31].

Epithelial and Lymphoid Lineage Markers

The neoplastic epithelial cells driving a thymoma will consistently express broad-spectrum epithelial markers, most notably the cytokeratin cocktail AE1/AE3, alongside CK5/6 and CK14. Furthermore, these neoplastic cells consistently express critical nuclear transcription factors, including p63, p40, and PAX8. The reactive, non-neoplastic T-lymphocytes swarming within the tumor stroma will stain highly positive for immature T-cell markers, including terminal deoxynucleotidyl transferase (TdT), CD1a, CD3, and CD99 [29]. Conversely, if the tumor represents a highly aggressive thymic carcinoma, the stroma will frequently lack these immature TdT-positive lymphocytes, instead presenting with fully mature T-cells and significant cytological atypia. Mature B-cells and plasma cells are exceedingly rare in conventional thymomas but may be highlighted with CD20 staining in specific, rare cases involving profound B-cell lymphoid hyperplasia [31].

Architectural Disruption and Epithelial Clustering

The most definitive histopathological feature distinguishing true, benign thymic hyperplasia from a Type B1 or B2 thymoma lies precisely in the architectural distribution of the cytokeratin-positive epithelial cells [33].

In a normal or actively hyperplastic thymus undergoing robust thymopoiesis, the thymic epithelial cells are widely separated from one another [33]. When stained with a pan-cytokeratin marker like AE1/AE3 or CK14, these benign epithelial cells exhibit extraordinarily long, thin, delicate cellular processes that reach out extensively to interact directly with the developing thymocytes. Under low-power microscopic magnification, this creates a highly characteristic, delicate, continuous three-dimensional network described extensively in the pathology literature as a "light and lacy pattern". The presence of this continuous lacy pattern confirms beyond doubt that the normal organotypic organization of the thymus is fully intact and functioning, robustly supporting a diagnosis of benign hyperplasia [32].

Conversely, in a true thymoma, this critical architectural integrity is entirely disrupted and lost [32]. While a well-differentiated Type B1 thymoma may attempt to mimic normal histology by displaying vague areas of medullary differentiation (referred to as medullary islands), the neoplastic epithelial cells fundamentally fail to form the delicate, interconnected lacy network. Instead, the cytokeratin-positive neoplastic cells display abnormal, cohesive aggregation. The presence of distinct epithelial clusters, defined rigorously under the microscope as three or more cohesive, contiguous epithelial cells gathered directly together without intervening lymphocytes, is a hallmark pathological feature. The identification of these cohesive clusters immediately shifts the definitive diagnosis away from benign hyperplasia and firmly toward a neoplastic B-type thymoma, regardless of the overall volume of lymphocytes present in the background (Table 5) [31].

**Table 5:** Definitive histopathological differentiation via immunohistochemistry profiling References:  [29,31,33]

Critical Histological Feature	Benign Thymic Hyperplasia	Lymphocyte-Rich Thymoma (WHO Type B1/B2)
Overall Organotypic Organization	Fully Intact (Clear normal cortex/medulla differentiation)	Severely distorted, entirely lost, or effaced by fibrosis
Cytokeratin Architecture (AE1/AE3)	"Lacy pattern" (delicate, continuous 3D network)	Clustered pattern (Cohesive nesting of contiguous cells)
Epithelial Cell Morphology	Widely spaced, featuring long, thin dendritic cellular processes	Neoplastic, cohesive, abnormally rounded, or distinctly polygonal
Background Lymphocyte Population	Heterogeneous mix of immature and mature reactive T-cells	Overwhelming dominance of immature T-cells (TdT+, CD1a+)
Presence of Hassall's Corpuscles	Universally present and morphologically normal	May be sparsely present in Type B1, generally absent in all others

The clinical management algorithm and ITMIG guidelines

Successful clinical management of an anterior mediastinal mass discovered in a patient with active GD requires a highly structured, patient-tailored, multidisciplinary approach involving seamless coordination between endocrinology, thoracic surgery, radiology, and pathology [18]. The ITMIG, working in conjunction with the European Society for Medical Oncology (ESMO), dictates guidelines ensuring that clinical management must be heavily tailored to both the radiological appearance of the lesion and the acute endocrine status of the patient [34].

The Severe Hazard of Premature Surgical Intervention

The single most critical clinical directive in this diagnostic scenario is the strict avoidance of premature, unnecessary invasive procedures. Subjecting a patient with active, medically uncontrolled GD to the profound physiological stress of general anesthesia, a core needle biopsy, or a minimally invasive thoracoscopic thymectomy carries an unacceptably high risk of triggering a catastrophic thyroid storm [7,21].

Thyroid storm is a rare, extreme, and life-threatening manifestation of thyrotoxicosis characterized by severe hyperpyrexia, profound cardiovascular collapse, tachyarrhythmias, altered mental status, and a mortality rate approaching thirty percent if not rapidly identified and treated. The acute physical trauma of thoracic surgery, combined with the immense pharmacological stress of anesthesia induction, acts as a profound precipitating trigger for this endocrine emergency [7,10].

Therefore, a rigid clinical rule must be applied: unless the mediastinal mass is causing critical, life-threatening airway compromise or severe vascular compression (such as impending Superior Vena Cava syndrome), all invasive diagnostic biopsies and therapeutic surgical resections must be strictly and unequivocally deferred until the patient's hyperthyroidism has been completely medically controlled and a stable euthyroid state achieved [5,16].

The Stepwise Clinical Management Pathway

Based on current oncological and endocrinological best practices, the clinical pathway for managing an incidental anterior mediastinal mass in a newly diagnosed GD patient proceeds through a rigorous stepwise algorithm:

Initial radiological stratification and triage: The initial contrast-enhanced CT or MRI must be meticulously reviewed by a specialized thoracic radiologist. If the mass exhibits overtly aggressive or malignant radiological features, such as massive size, dense macro-calcifications, areas of cystic necrosis, indistinct invasive borders, or frank invasion into the pericardium, pleura, or adjacent great vessels, the clinical suspicion for a true thymic carcinoma or an advanced invasive thymoma is exceedingly high. In such high-risk cases, the patient must be aggressively treated with high-dose antithyroid medications, beta-blockers, and potentially glucocorticoids to achieve rapid euthyroidism. Once euthyroid, the patient is immediately fast-tracked for an image-guided biopsy or definitive surgical resection to prevent further oncological spread [5,7].

Implementation of a conservative "wait and see" policy: If the initial imaging demonstrates a highly homogeneous mass with perfectly preserved fat planes and no invasive features, a dedicated chemical shift MRI should be immediately obtained [7]. If the chemical shift MRI yields a SII greater than 9% and a CSR less than 0.7, a firm, presumptive diagnosis of benign TH secondary to GD is made [22]. At this juncture, the patient is commenced on standard medical therapy for GD (e.g., methimazole or propylthiouracil) and an expectant, conservative "wait and see" policy is formally adopted, with absolutely no thoracic surgical intervention recommended [23].

Mandatory longitudinal radiographic tracking: A follow-up, high-resolution CT or MRI is rigorously scheduled for exactly three to six months post-initiation of antithyroid therapy to track volumetric changes [16]. If the six-month follow-up imaging reveals a significant volumetric regression of the mass by 50% or more, the diagnosis of benign TH secondary to GD is definitively confirmed. The patient safely continues routine endocrine management, with an expected full, structural resolution of the mediastinal mass by the 12-month follow-up interval [16]. If the mass demonstrates indolent growth, or crucially, fails to achieve at least a 50% volumetric regression despite the proven normalization of thyroid function panels and the reduction of TSH-R autoantibodies, the conservative tracking approach must be immediately abandoned [16].

Definitive surgical resection: For non-involuting, medically resistant masses, complete surgical resection is strongly favored over core needle biopsy. As previously established, small biopsies of the prevascular compartment carry severe risks of both diagnostic sampling error and physical capsular breach, the latter of which carries a potential risk of tumor seeding if the lesion happens to be an early-stage thymoma [35]. ITMIG strongly recommends employing minimally invasive surgical approaches, such as video-assisted thoracic surgery (VATS) or robotic-assisted thoracic surgery (RATS), for well-circumscribed lesions measuring under 5 cm. These advanced robotic and video-assisted approaches significantly reduce postoperative pain, vastly accelerate patient recovery times, and provide definitive, complete histological staging and curative resection without the profound morbidity of a traditional median sternotomy [34].

While the proposed algorithm advocates for conservative management, specifically achieving euthyroidism followed by repeat imaging, clinical reality often necessitates flexibility. In this case, the patient was extensively counseled on the high likelihood of benign TH and the recommended conservative follow-up. However, the incidental finding provoked significant anxiety regarding a potential malignancy. Prioritizing patient autonomy and shared decision-making, a mutual agreement was made to proceed with an early core biopsy for definitive diagnostic certainty and peace of mind, deviating from the initial watchful waiting strategy.

## Conclusions

The discovery of an anterior mediastinal mass in a patient with GD presents a critical clinical dilemma, as distinguishing benign TH from a true thymic epithelial neoplasm, such as a thymoma, is paramount. Because premature surgical intervention or invasive biopsy carries a profound risk of precipitating a life-threatening thyroid storm, a conservative, medically managed approach is preferred.

Chemical shift MRI serves as a valuable, non-invasive diagnostic modality for differentiating these entities by identifying the microscopic fat characteristic of hyperplasia, which is notably absent in solid tumors. When tissue sampling is ultimately required, clinicians and pathologists must recognize the severe limitations of small CNBs. Definitive histopathological diagnosis relies heavily on broad architectural evaluation and immunohistochemistry to distinguish the benign "lacy pattern" of hyperplasia from the cohesive epithelial clustering indicative of a thymoma. Ultimately, initial management must prioritize the achievement of a stable euthyroid state and the utilization of longitudinal imaging to monitor for mass regression, reserving definitive surgical resection strictly for radiologically aggressive or medically resistant, non-involuting lesions.
